# Sleep disorders in Parkinson’s disease, an early and multiple problem

**DOI:** 10.1038/s41531-024-00642-0

**Published:** 2024-02-29

**Authors:** Pauline Dodet, Marion Houot, Smaranda Leu-Semenescu, Jean-Christophe Corvol, Stéphane Lehéricy, Graziella Mangone, Marie Vidailhet, Emmanuel Roze, Isabelle Arnulf

**Affiliations:** 1 Service des Pathologies du Sommeil et Centre de Référence National des Narcolepsies et Hypersomnies rares, Assistance Publique-Hôpitaux de Paris-Sorbonne (AP-HP-Sorbonne), Hôpital la Pitié-Salpêtrière, Paris, France; 2 Paris Brain Institute (ICM), Sorbonne University, Inserm U1227, CNRS 7225 Paris, France; 3 Center of Excellence of Neurodegenerative Disease (CoEN), AP-HP, Pitié-Salpêtrière Hospital, Paris, France; 4 Department of Neurology, Institute of Memory and Alzheimer’s Disease (IM2A), AP-HP, Pitié-Salpêtrière Hospital, Paris, France; 5 Assistance Publique Hôpitaux de Paris, Inserm, Clinical Investigation Centre (CIC) Neuroscience, Paris Brain Institute - ICM, Pitié-Salpêtrière Hospital, Paris, France; 6 Assistance Publique Hôpitaux de Paris, Hôpital Pitié -Salpêtrière, Department of Neuroradiology, 75013 Paris, France

**Keywords:** Parkinson's disease, Neurological manifestations

## Abstract

In Parkinson’s disease (PD), it remains unclear whether sleep disorders including insomnia, REM sleep behavior disorder (RBD), excessive daytime sleepiness (EDS), restless legs syndrome (RLS) and sleep-disordered breathing (SDB), are isolated or combined, interact with each other and are associated with clinical factors. We sought to determine the prevalence and combinations of the main sleep disorders, and their clinical and polysomnographic associations in early stage PD. Sleep disorders were systematically diagnosed after medical interview and video-polysomnography in 162 participants with early stage PD and 58 healthy controls from the baseline of the longitudinal ICEBERG cohort. Demographic, clinical (motor, cognitive, autonomic, psychological and sensory tests), therapeutic and polysomnographic associations of sleep disorders were investigated. Sleep disorders were frequent (71%) and combined in half of the patients. The number of sleep disorders increased with disease duration and dysautonomia. Insomnia was the most common (41%), followed by definite RBD (25%), EDS (25%), and RLS (16%). These disorders were more frequent than in controls whereas SDB was rare, moderate and similar in both groups. In patients, insomnia (mainly difficulties maintaining sleep) was associated with female gender, shorter sleep time and RLS, but not with motor or psychological symptoms. RBD was associated with dysautonomia and advanced age, but not with motor and cognitive measures. EDS was associated with psychiatric and motor symptoms as well as the sedative effects of dopamine agonists but not with other sleep disturbances. Sleep disturbances are frequent and combined in early patients with PD. Their determinants and markers are more organic than psychological.

## Introduction

Altered sleep is common in Parkinson’s disease (PD), and negatively impacts the quality of life of patients and their caregivers^1^. Sleep disturbances include insomnia, violent behaviors during the night often corresponding to rapid eye movement sleep behavior disorder (RBD), excessive daytime sleepiness (EDS), restless legs syndrome (RLS), and sleep-disordered breathing (SDB)^1,2^. Most of these sleep problems are worsened by comorbid psychiatric disorders (including anxiety and mood disorders)^3^, dopaminergic treatment^4,5^,motor symptoms and disease duration^6^. Sleep disorders are already present in prodromal^7–9^ and the early stage of PD^6,10^ and their frequency increases progressively from the onset of the disease^3,4,6,11^. They can be isolated or combined, thus interacting with each other^6^. Despite the importance of considering the complexity of their combination, most studies have described each sleep disturbance separately^4,5,11^ or in the context of a broader range of nonmotor symptoms^12,13^. Only two studies (questionnaire-based only) examined the interaction between insomnia, clinical RBD as assessed by questionnaires, and EDS (defined as Epworth Sleepiness Scale (ESS) score > 10/24) (but not RLS)^6,14^, only one of which examined early stage PD^6^. Neither used polysomnography, which is essential for diagnosing SDB and confirming RBD.

The aims of this study were to determine the prevalence of sleep disturbances (insomnia, EDS, definite RBD, RLS, and SDB) in a large, systematic series of participants at an early stage of PD enrolled in a cohort for predicting PD trajectories, and in healthy controls (both studied with video-polysomnography), with a special emphasis on combination of sleep disturbances, as well as their clinical and polysomnographic associations.

## Results

### Demographic and clinical characteristics of the groups

Of the 168 PD participants and 60 controls who took part in the study, 6 PD participants and 2 controls were excluded due to missing sleep recordings (*N* = 7) and exclusion criteria (*N* = 1), bringing to 162 the number of PD participants and 58 the number of healthy controls included in this analysis. The demographic and clinical characteristics of participants with PD and controls are shown in Table 1. The age at onset of PD symptoms was 59.4 ± 9.3 years and age at diagnosis was 61 ± 9.1 years. The disease course was 18.1 ± 13.1 months. The total LED dose/24 h was 290 ± 257 mg. Scores were worse in the PD group than in the control group for motor, nonmotor, cognitive, and mood/behavioral scales, with the exception of MoCA scores, which were not different. The percentage of participants treated with psychotropic medications did not differ between groups, including benzodiazepines (4 [2.5%] in the PD group *vs*. 3 [6%] in the control group, *P* = 0.4), melatonin (14 [9%] *vs*. 1 [2%], *P* = 0.12), hypnotics (1 [1%] *vs*. 0 [0%], *P* = 1), and antidepressants (13 [8%] *vs*. 2 [4%], *P* = 0.4).

**Table 1:** Demographical and clinical characteristics of participants with early stage PD and healthy participants

	PD	Controls	*p*^a^	MDE or OR ± SE	Adjusted *p*^b^	Corrected *p*^c^
**Number of participants**	162	58		–	–	–
**Demographic data**						
Age at polysomnography (y)	62.4 ± 9.2	61.9 ± 9.2	0.72	–	–	–
Male sex, *N* (%)	103 (64%)	30 (51%)	0.09	–	–	–
Body mass index (weight/m^2^)	25.2 ± 3.8	25.5 ± 4.3	0.69	−0.38 ± 0.6	0.53	0.53
**Ethnicities (self defined)**						
Caucasian origin, *N* (%)	144 (89%)	54 (93%)	0.83	–	–	–
African origin, *N* (%)	14 (9%)	3 (5%)	–	–	–	–
Asian origin, *N* (%)	1 (0.6%)	4 (2%)	–	–	–	–
**Motor manifestations**						
MDS-UPDRS						
Part I	9.3 ± 4.0	5.0 ± 3.4	**<0.001**	4.22 ± 0.6	**<0.001**	**<0.001**
Part II	8.2 ± 3.8	1.2 ± 1.8	**<0.001**	6.83 ± 0.5	**<0.001**	**<0.001**
Part III OFF	29.7 ± 7.8	5.4 ± 5.1	**<0.001**	23.81 ± 1.1	**<0.001**	**<0.001**
Total	47.2 ± 11.9	11.5 ± 8.4	**<0.001**	34.99 ± 1.6	**<0.001**	**<0.001**
Axial motor score	2.9 ± 1.9	1.0 ± 1.4	**<0.001**	1.83 ± 0.3	**<0.001**	**<0.001**
**Non motor manifestations**						
HAD-Anxiety (0–21)	6.9 ± 3.2	5.9 ± 2.6	**0.002**	1.14 ± 0.5	**0.02**	**0.02**
HAD-Depression (0–21)	3.8 ± 2.9	2.2 ± 3.0	**<0.001**	1.55 ± 0.5	**0.001**	**0.002**
Mini-SEA score total	33.2 ± 5.1	35.2 ± 3.9	**0.003**	1.86 ± 0.7	**0.007**	**0.01**
Starkstein Apathy Scale	9 ± 4.8	6.9 ± 3.1	**<0.001**	−0.92 ± 0.4	**0.02**	**0.03**
Cognition (MoCA, 0–30)	27.5 ± 2.0	28.1 ± 1.8	**0.005**	−0.49 ± 0.3	0.10	0.11
Frontal assessment battery (0–18)	16.4 ± 1.7	17.0 ± 1.1	**0.002**	−0.51 ± 0.2	**0.03**	**0.04**
SCOPA-AUT	12.3 ± 7.4	7.4 ± 6.1	**<0.001**	4.77 ± 1.0	**<0.001**	**<0.001**
Drop in SBP (mmHg)	2.9 ± 10.8	−2.27 ± 10.3	**0.002**	4.54 ± 1.6	**0.006**	**0.01**
Orthostatic drop in SBP (>10 mmHg)^d^	34 (21%)	5 (8%)	**0.03**	2.69 ± 1.4	**0.04**	**0.04**
Non Motor Symptoms Scale	8.2 ± 4.6	3.6 ± 3.7	**<0.001**	4.54 ± 0.7	**<0.001**	**<0.001**
Olfaction (UPSIT, 0–40)	22.0 ± 6.5	32.5 ± 5.7	**<0.001**	10.15 ± 0.9	**<0.001**	**<0.001**

Data are given as mean ± standard deviation (for continuous measures) or *N* (%) (for categories). Mean difference estimated (MDE) and Odds ratio ± Standard error (OR ± SE) were extracted from Generalized Linear model (GLM) adjusted for age and sex, using with Gaussian family and identity link for numerical measures and using Bernoulli family and logit link for binary measures.

Statistical significance was set at *p* < 0.05. Significant differences are shown in bold.

*HAD* Hospital Anxiety and Depression scale, *MDS-UPDRS* Movement Disorder Society-Sponsored Revision of Unified Parkinson’s Disease Rating Scale, *Mini-SEA* Mini Social and Emotional Assessment, *MoCA* Montreal Cognitive Assessment, *PD* Parkinson’s disease, *SBP* systolic blood pressure, *SCOPA-AUT* Scales for Outcomes in Parkinson’s Disease—Autonomic dysfunction, *UPSIT* University of Pennsylvania Smell Identification Test (40-item version), *MDE* Mean difference estimated, *SE* standard error, *OR* Odds ratio.

^a^*p* values from Fisher’s exact test for binary measures or Welsch’s *t* tests for numerical measures.

^b^*p* values from GLM adjusted for age and sex otherwise.

^c^*p* values corrected for multiple testing using the Benjamini–Hochberg procedure.

^d^GLM using Bernoulli family and logit link for binary measures and thus OR were given in the MDE or OR ± SE column.

Statistical significance was set at *p* < 0.05. Significant differences are shown in bold.

### Sleep disorders in Parkinson’s disease compared to control group

In the PD group, 41% [65/162] of participants had insomnia, with an odds ratio (OR) almost three times higher than in the control group ([12/58], 21%) after adjustment for age, sex and apnea-hypopnea index (Table 2 and supplementary Fig. 1). Difficulties maintaining sleep (not initiating it) characterized the PD group compared to the control group ([56/162], 35% versus 16% [9/162]). Participants with PD ([40/162], 25%) were more likely than controls ([5/58], 8%) to have EDS (defined as ESS > 10). Disorders of arousal were rare in both groups, and video-polysomnography-defined RBD was found exclusively in participants with PD (([41/162], 25%) but not in controls ([0/58], 0%). Restless leg syndrome affected 16% ([25/162]) of participants with PD, a frequency that tended to be higher than in controls ([3/58], 5%) although not significant. The percentage of participants with SDB, regardless of the threshold chosen for the apnea-hypopnea index, was low ([19/162], 12% of participants with PD had more than 15 apneas or hypopneas per hour of sleep and was not different compared to controls ([11/58], 19%).

**Table 2:** Baseline comparisons of sleep disturbances and sleep recordings between PD and controls groups

	PD patients *N* = 162	Controls *N* = 58	*p*^a^	MDE or OR ± SE	Adjusted *p*^b^	Corrected *p*^c^
**Sleep disturbances**						
*Insomnia*						
All types	65 (41%)	12 (21%)	**0.01**	OR = 2.91 ± 1.09	**0.002**	**0.02**
Difficulty into initiating sleep	21 (13%)	8 (14%)	0.80	OR = 1.05 ± 0.48	0.87	1.00
Difficulty into maintaining sleep	56 (35%)	9 (16%)	**0.01**	OR = 3.22 ± 1.33	**0.003**	**0.02**
Both difficulties	13 (8%)	5 (9%)	0.80	OR = 1.04 ± 0.58	0.89	1.00
*Hypersomnolence*						
Excessive daytime sleepiness	40 (25%)	5 (8%)	**0.01**	OR = 3.27 ± 1.65	**0.01**	**0.04**
Score at the Epworth sleepiness scale (0–24)	7.6 ± 4.1	6.1 ± 3.3	**0.01**	MDE = 1.44 ± 0.60	**0.02**	0.06
*Parasomnia*						
Disorders of arousal (non-REM parasomnia)^d^	9 (6%)	1 (2%)	0.46	-	-	-
V-PSG defined REM sleep behavior disorder^d^	41 (25%)	0 (0%)	**<0.001**	-	-	-
RBDQ-HK total score^e^	12.2 ± 13.2	8.3 ± 7.5	**0.05**	MDE = 0.56 ± 0.27	0.38	0.57
Factor 1 of RBDQ-HK (dream-related)	5.5 ± 5.5	4.8 ± 3.7	0.36	MDE = 0.71 ± 0.82	**0.02**	0.06
Factor 2 of RBDQ-HK (behavioral manifestation)^e^	6.7 ± 9	3.5 ± 5	**0.02**	MDE = 0.56 ± 0.27	0.17	0.31
V-PSG, REM sleep without atonia (%, any)	20.7 ± 26	3.4 ± 9.9	**<0.001**	MDE = 16.79 ± 3.64	**<0.001**	**<0.001**
*Sleep-related movement disorders*						
Restless legs syndrome (RLS)^d^	25 (16%)	3 (5%)	0.06	-	-	-
Periodic leg movements during sleep (n/h)^e^	7.7 ± 20.2	13.3 ± 21	0.08	MDE = −1.08 ± 0.35	**0.006**	**0.026**
Periodic leg movements during sleep ( > 15)	22 (14%)	14 (25%)	0.07	OR = 0.39 ± 0.17	**0.05**	0.11
*Sleep-disordered breathing*						
Apneas-hypopneas index (n/h)^e^	6.4 ± 8.1	7.8 ± 9.0	0.26	MDE = −0.38 ± 0.22	0.08	0.16
Apnea-hypopnea index >15	19 (12%)	11 (19%)	0.18	OR = 0.46 ± 0.20	0.07	0.14
Apnea-hypopnea index = 15–30	14 (9%)	7 (12%)	0.44	OR = 0.59 ± 0.30	0.96	1.00
Apnea-hypopnea index > 30^d^	5 (3%)	4 (7%)	0.24	-	-	-
*Co-occurrence of sleep disturbances*						
No sleep disturbance	46 (29%)	32 (58%)	**<0.001**	OR = 3.52 ± 0.31	**<0.001**	**<0.001**
One sleep disturbance	58 (36%)	17 (31%)				
More than one sleep disturbance	55 (35%)	6 (11%)				
**Sleep night**						
Total sleep time (min)	364.7 ± 67.3	368.8 ± 80.3	0.70	MDE = −0.23 ± 10.55	0.97	1.00
Sleep efficiency (%)	79.5 ± 11.1	80.7 ± 12.6	0.50	MDE = − 0.59 ± 1.71	0.69	0.93
Wake after sleep onset (min)	94.7 ± 53.3	85.7 ± 50.4	0.27	MDE = 6.42 ± 7.83	0.37	0.57
**Latency to (min)**						
Sleep onset^e^	19.2 ± 18.7	19.4 ± 18.2	0.93	MDE = 0.02 ± 0.29	0.95	1.000
REM sleep	148.4 ± 87	103.8 ± 64.8	**<0.001**	MDE = 45.75 ± 12.74	**<0.001**	**0.003**
**Sleep stages, % of total sleep time**						
N1^e^	6.2 ± 5.5	4.1 ± 3.9	**0.008**	MDE = 0.33 ± 0.16	**0.02**	0.06
N2	52.6 ± 12	51.5 ± 10	0.53	MDE = 0.95 ± 1.77	0.49	0.7
N3	25.3 ± 10.2	25.8 ± 9.5	0.74	MDE = 0.02 ± 1.50	0.83	1.00
REM sleep	16 ± 8	18.7 ± 6.8	**0.03**	MDE = −2.66 ± 1.18	**0.03**	0.06
**Sleep fragmentation**						
Fragmentation index (n/h)	12.4 ± 6.9	15 ± 8.8	**0.02**	MDE = −2.82 ± 1.14	0.08	0.16
Apneas-hypopneas index (n/h)^e^	6.4 ± 8.1	7.8 ± 9.0	0.26	MDE = −0.38 ± 0.22	0.08	0.16
Apneas-hypopneas index < 15/h	143 (88%)	46 (81%)	0.18	OR = 2.19 ± 0.96	1.00	1.00
Periodic leg movements (n/h)^e^	7.7 ± 20.2	13.3 ± 21	0.08	MDE = −1.08 ± 0.35	**0.006**	**0.03**

Data are given as mean ± SD (for numerical variables) or *N* (%) (for categorical variables).

Mean difference estimated (MDE) and Odds ratio Standard error (OR ± SE) were extracted from Generalized Linear model (GLM) adjusted for age, sex, and apneas-hypopneas index, using with gaussian family and identity link for numerical measures and using Bernoulli family and logit link for binary measures.

Statistical significance was set at *p* < 0.05. Significant differences are shown in bold.

The fragmentation index was the sum of number of wakes and arousals per hour divided by the total sleep time.

*HC* Healthy Controls, *MDE* Mean difference estimated, *OR* Odds ratio, *PD* Parkinson’s disease, *RBD* rapid eye movement sleep (REM) behavior disorder, *SE* standard error.

^a^*p* values from Fisher’s exact test for binary measures or Welsch’s *t* tests for numerical measures.

^b^*p* values from GLM adjusted for age, sex and apneas-hypopneas index except for the variable apneas-hypopneas index.

^c^*p* values corrected for multiple testing using Benjamini–Hochberg procedure.

^d^Binary measures with group frequencies < 5 for which only a Fisher’s exact test was performed to compare clinical groups due to lack of statistical power to use GLM.

^e^Measure were square root transformed, as rightskewed, before GLM was performed.

### Video-polysomnography measures

On video-polysomnography (Table 2), total sleep time, sleep efficiency, duration of wakefulness after sleep onset and fragmentation index, as well as N2 and N3 sleep percentages were not different between the PD and control groups after adjustment for age, gender, and apnea-hypopnea index. In the PD group, REM sleep latency was longer, the percentage of N1 tended to be higher, and REM sleep percentage tended to be lower than in the control group. By restricting the analysis to participants not taking antidepressants (sensitivity analysis), the differences between the groups for N1 and REM sleep percentages disappeared (data not shown) but the difference for REM sleep latency remained the same. The percentage of REM sleep without atonia was higher in the PD group than in the control group. The PLM index was higher in the control group than in the PD group. Abnormal ( > 15/h) PLM during sleep tended to be more frequent in the control group than in the PD group, however the significance disappeared after correction. The apnea-hypopnea index did not differ between groups.

### Combinations of sleep disturbances in PD

**Fig. 1:**
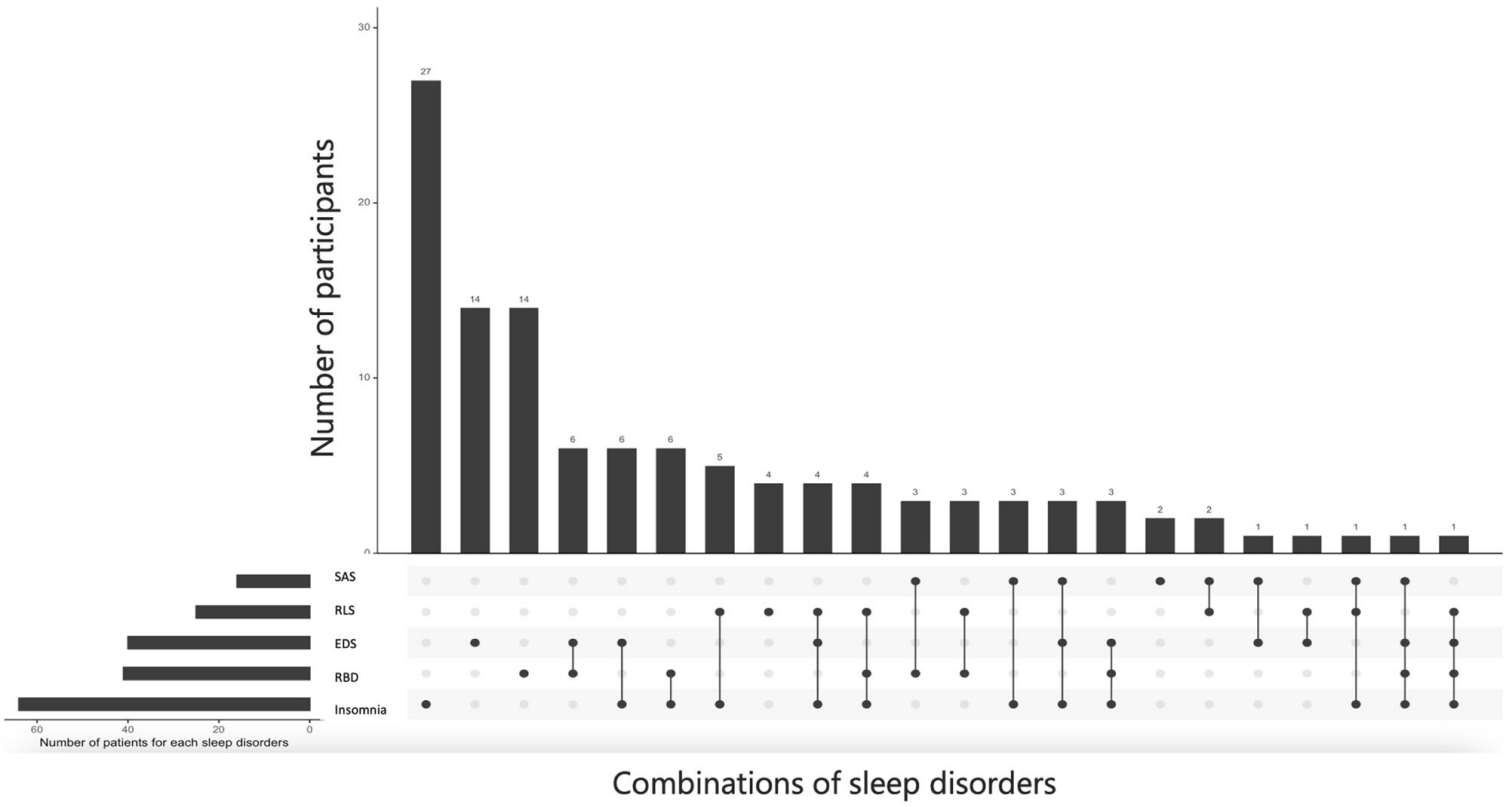
Combined sleep disturbances in participants with Parkinson’s disease. Each vertical bar represents the number of patients corresponding to the combined disorders indicated by the connected dots below. Each horizontal bar represents the total number of patients with a specific sleep disorder. RBD: REM behavior disorder; EDS excessive daytime sleepiness; RLS Restless legs syndrome; SDB sleep-disordered breathing.

Among PD participants, 113/162 (72%) had at least one sleep disturbance compared to 23/58 (42%) among controls. PD participants had more frequent co-occurrence of sleep disturbances (>1 sleep disturbance in 55/162 (35%) cases) compared to controls (6/58 (11%)) (Table 2). Among PD participants, 17/162 (11%) had more than two sleep disturbances. Combined sleep disturbances in PD participants are presented in Fig. 1. Isolated insomnia was the most common, followed by isolated EDS and isolated RBD. Combinations of disturbances were scattered across the group, as each sleep disorder could be associated with any other. PD participants with insomnia had another sleep disorder in 58% (37/64) of cases, with no preference other than RLS. PD participants with EDS and RBD had another sleep disorder in 65% (26/40) and 66% (27/41) of cases, respectively, with no preference.

Demographic and clinical characteristics by co-occurrence of sleep disorders in PD participants are presented in Table 3, depending on presenting no sleep disorder, a single one or multiple sleep disorders. PD participants with multiple sleep disorders were older at PSG time and tended to have longer disease duration than those without sleep disorder. Modified MDS-UPDRS part 1 (excluding sleep-related items 1.7 and 1.8) was higher in groups with a single sleep disorder and with multiple sleep disorders compared with participants without sleep disorder. The scores at MDS-UPDRS part 2 and total MDS-UPDRS were higher in the group with multiple sleep disorders, whereas MDS-UPDRS part 3 scores were similar in the three groups. The total HADS score was higher in groups with than without sleep disorders. PD participants with multiple disorders had higher scores of dysautonomia (drop in systolic blood pressure, orthostatic drop, SCOPA-AUT, NMSS), but no lower cognitive performance. A forward stepwise procedure using the AIC criterion was used to select the measures most associated with an increase in the number of sleep disturbances using ordinal logistic regression. Disease duration in months (OR = 1.02 [1.00, 1.05], *p* = 0.037), modified MDS-UPDRS part 1 (OR = 1.12 [1.01, 1.25], *p* = 0.028), and SCOPA-AUT score (OR = 1.06 [1.00, 1.11], *p* = 0.034) were associated with co-occurrence of multiple sleep disturbances in this model.

**Table 3:** Demographical and clinical characteristics by co-occurrence of sleep-related disturbances in PD participants

Group	No sleep disturbance^a^ *n* = 46 (28%)	One sleep disturbance^b^ *n* = 58 (36%)	Multiple sleep disturbances^c^ *n* = 55 (34%)	*p*
**Demography**				
Age at PSG (y)	60.7 [49.5, 67.6]	63.0 [58.1, 70.0]	64.9 [60.9, 70.4]	**0.047**
Disease duration (months)	12.9 [6.6, 20.3]	12.6 [5.4, 28.4]	18.1 [9.9, 31.2]	0.06
Male sex, (*N* (%)	30 (65%)	33 (57%)	39 (71%)	0.32
Body mass index (weight/m^2^)	25.2 [22.9, 28.0]	24.5 [22.6, 27.0]	25.5 [23.2, 28.3]	0.38
**Motor manifestations**				
Modified MDS-UPDRS Part I	4.0 [3.0, 6.0] ^b, c^	6.5 [5.0, 9.0] ^a^	8.0 [6.0, 10.0] ^a^	**<0.001**
MDS-UPDRS Part II	6.0 [4.0, 9.8] c	8.0 [5.0, 11.0]	10.0 [7.0, 11.5] ^a^	**0.002**
MDS-UPDRS Part III OFF	28.0 [25.0, 35.5]	29.0 [23.2, 33.8]	31.0 [26.5, 35.5]	0.38
MDS-UPDRS Total (0–142)	39.5 [36.0, 51.0] ^c^	47.5 [40.0, 55.0] ^c^	52.0 [41.5, 60.5] ^a, b^	**0.001**
Axial motor score	2.0 [1.0, 4.0]	3.0 [1.0, 4.0]	3.0 [2.0, 4.0]	0.48
**Non motor manifestations**				
HAD-Anxiety (norm<7)	6.0 [3.0, 8.0] ^b^	7.0 [5.2, 9.8] ^a^	6.0 [5.0, 9.0]	**0.013**
HAD-Depression (norm<7)	2.0 [1.0, 4.0] ^c^	3.0 [2.0, 5.8]	5.0 [2.0, 7.0] ^a^	**0.018**
HAD-Total (0–21)	8.5 [6.0, 12.0] ^b, c^	10.5 [7.2, 14.0] ^a^	11.0 [7.5, 16.0] ^a^	**0.007**
Starkstein Apathy Scale (0–42, norm<15)	8.0 [5.0, 11.0]	8.5 [6.0, 11.8]	9.0 [6.5, 12.5]	0.23
Cognition (MoCA, 0–30)	28.0 [26.0, 29.0]	28.0 [26.0, 29.0]	28.0 [26.0, 29.0]	0.92
SCOPA-AUT	7.0 [5.0, 12.8] ^b, c^	11.0 [7.0, 15.8] ^a, c^	13.0 [9.0, 21.8] ^a, b^	**<0.001**
Supine drop in SBP, mmHg	−0.5 [−6.0, 3.0] ^c^	1.5 [−5.0, 6.5] c	6.0 [1.0, 12.0] ^a, b^	**<0.001**
Orthostatic SBP drop >10 mmHg	5 (11%) c	9 (16%) c	20 (37%) ^a,b^	**0.002**
Nonmotor Symptom Scale	6.0 [2.2, 8.0] ^b, c^	8.0 [5.0, 10.8] ^a, c^	11.0 [8.0, 14.0] ^a, b^	**<0.001**
Olfaction (UPSIT 0–40)	24.0 [18.0, 28.0]	22.0 [19.0, 26.5]	20.0 [16.0, 25.0]	0.11
**Treatment (daily dose), mg/d**				
Total levodopa equivalent	187.1 [100.0, 400.0]	300.0 [100.0, 400.0]	298.5 [162.1, 487.1]	0.41
Dopamine agonist levodopa equivalent	38.6 [0.0, 149.9]	0.0 [0.0, 111.4]	74.3 [0.0, 150.0]	0.30
Levodopa	0.0 [0.0, 200.0]	131.2 [0.0, 300.0]	50.0 [0.0, 299.6]	0.18

Data are given as mean ± SD or *N* (%). *P* = *p* values from Kruskal–Wallis test for numerical variables and Fisher’s exact test for categorical variables. Statistical significance was set at *p* < 0.05. Significant differences are shown in bold. To determine which groups differed from each other, post hoc comparisons were made using the pairwise Mann–Whitney–Wilcoxon test for numerical variables and the pairwise Fisher’s exact test for categorical variables, both followed by the Benjamini–Hochberg correction to account for multiple testing.

Modified MDS-UPDRS part I mean without the items 1.7 and 1.8. *HAD* Hospital Anxiety and Depression scale, *MDS-UPDRS* Movement Disorder Society-Sponsored Revision of Unified Parkinson’s Disease Rating Scale, *MoCA* Montreal Cognitive Assessment, *NMSS* Non-Motor Symptoms Scale, *SBP* systolic blood pressure, *SCOPA-AUT* Scales for Outcomes in Parkinson’s Disease—Autonomic dysfunction; UPSIT, University of Pennsylvania Smell Identification Test (40-item version).

^a^For a pairwise difference with no sleep disturbance.

^b^For a pairwise difference with one sleep disturbance.

^c^For a pairwise difference with multiple sleep disturbances.

### Factors associated with insomnia

Within the PD group, the demographical, clinical and sleep characteristics according to the presence of insomnia, EDS, RBD, RLS and SDB are shown in Tables 4 and 5. Participants with insomnia ([65/160], 41% ; missing data = 2) were more often women and had a lower body mass index but had no difference in age and disease duration compared to PD participants without insomnia. Insomnia in PD was associated with higher disability scores at several nonmotor scales including Part I of the MDS-UPDRS, NMSS, and SCOPA-AUT, but not with motor scores (Part II, motor examination in off-dopamine treatment [Part III], and axial motor score) or dosage of dopaminergic and levodopa treatments, scores at psychiatric scales (anxiety, depression and apathy) scales, cognitive and olfactive performances, and orthostatic hypotension. In terms of sleep, insomnia was associated with higher scores at Factor 1 (dream changes) of RBDQ-HK (but not with definite RBD), more frequent RLS (but P = 0.05), and shorter total sleep time and sleep efficiency (but not with sleep latencies, percentage of sleep stages and fragmentation index) during video-polysomnography, but not with EDS, SDB and abnormal PLM index.

**Table 4:** Baseline clinical characteristics depending on sleep-related disturbances in PD participants

	Insomnia (*N* = 160)		*p*	Excessive daytime sleepiness (*N* = 161)		*p*	Define RBD (*N* = 160)		*p*	Restless leg syndrome (*N* = 160)		*p*
Group	No (*n* = 95)	Yes (*n* = 65)		No (*n* = 121)	Yes (n = 40)		No (*n* = 119)	Yes (*n* = 41)		No (*n* = 135)	Yes (*n* = 25)	
**Demography**												
Age at PSG (y)	63.3 [56.0;69.5]	63.8 [58.6;69.7]	0.42	64.5 [56.6;70.2]	62.4 [55.9;68.8]	0.43	62.2 [54.8;68.7]	67.2 [60.8;71.5]	**0.005**	63.3 [56.0;69.7]	64.9 [60.9;70.0]	0.33
Disease duration (months)	14.6 [7.05;27.6]	14.2 [6.9;29.5]	0.97	14.5 [7.0;27.8]	16.1 [6.88;31.2]	0.45	13.1 [6.25;26.3]	19.0 [10.9;29.5]	0.16	12.8 [6.45;25.4]	31.6 [22.3;36.0]	**<0.001**
Male sex, (*N* (%)	67 (71%)	35 (54%)	**0.04**	47 (39%)	11 (28%)	0.26	71 (60%)	30 (73%)	0.14	90 (67%)	12 (48%)	0.11
Body mass index (weight/m^2^)	25.6 [23.8;28.4]	24.2 [22.6;26.3]	**0.02**	24.8 [22.7;27.6]	25.4 [23.6;28.0]	0.45	24.4 [22.4;26.9]	26.4 [24.3;28.3]	**0.007**	25.2 [23.0;27.9]	24.2 [23.0;26.6]	0.45
**Motor manifestations**												
MDS-UPDRS Part I	8.0 [5.50;10.0]	10.0 [8.0;13.0]	**<0.001**	9.0 [6.0;11.0]	10.0 [8.0;13.0]	**0.006**	8.0 [6.0;11.5]	10.0 [8.0;13.0]	**0.004**	9.0 [6.00;11.0]	10.0 [9.00;13.0]	**0.02**
MDS-UPDRS Part II	8.0 [5.0;11.0]	8.0 [5.0;10.0]	0.73	7.0 [5.0;10.0]	10.0 [7.0;13.0]	**<0.001**	7.0 [5.0;10.0]	9.0 [7.0;11.0]	**0.001**	8.0 [5.0;10.0]	9.0 [7.0;11.0]	0.28
MDS-UPDRS Part III OFF	29.0 [25.0;37.0]	29.0 [24.0;33.0]	0.14	29.0 [24.0;34.0]	31.0 [27.0;36.3]	0.09	29.0 [24.5;34.5]	31.0 [23.0;37.0]	0.30	29.0 [25.0;35.0]	29.0 [22.0;35.0]	0.74
MDS-UPDRS Total (0–142)	47.0 [37.5;56.0]	48.0 [39.0;54.0]	0.98	44.0 [37.0;54.0]	52.0 [45.0;61.0]	**0.001**	45.0 [37.0;54.0]	50.0 [43.0;60.0]	**0.007**	47.0 [38.0;55.0]	50.0 [40.0;59.0]	0.25
Axial motor score	3.0 [2.00;4.00]	3.00 [1.0;4.00]	0.09	3.0 [1.0;4.00]	3.00 [2.0;4.00]	0.11	3.0 [1.0;4.0]	3.0 [2.0;4.0]	0.65	3.0 [1.0;4.0]	3.0 [1.0;5.0]	0.62
**Non motor manifestations**												
HAD-Anxiety	6.0 [4.0;8.00]	7.0 [5.0;9.00]	0.18	6.0 [4.0;8.0]	7.50 [6.0;9.0]	**0.04**	7.0 [4.0;9.0]	7.0 [5.0;8.0]	0.51	7.0 [5.0;9.0]	6.0 [5.0;9.0]	0.35
HAD-Depression	3.0 [1.0;6.00]	3.0 [2.0;7.00]	0.72	3.0 [1.0;5.0]	6.0 [2.75;7.25]	**<0.001**	3.0 [1.0;6.0]	5.0 [2.0;7.0]	**0.04**	3.0 [1.0;7.0]	4.0 [1.0;5.0]	0.93
HAD-Total (range :0–21, norms<7 for each part)	10.0 [7.0;14.0]	10.0 [7.0;15.0]	0.37	9.0 [6.0;13.0]	13.0 [9.0;16.0]	**<0.001**	9.0 [6.50;13.0]	12.0 [9.0;15.0]	0.05	10.0 [7.0;14.0]	9.0 [6.0;13.0]	0.53
Starkstein Apathy Scale (range :0–42, norm<15)	9.0 [5.0;11.5]	9.00 [6.0;11.0]	0.99	8.0 [5.0;11.0]	11.0 [7.75;14.0]	**0.004**	9.0 [5.0;11.0]	9.00 [6.0;13.0]	0.12	9.0 [6.0;11.0]	7.0 [4.0;11.0]	0.22
Cognition (MoCA, 0–30)	28.0 [26.0;29.0]	28.0 [26.0;29.0]	0.86	28.0 [26.0;29.0]	28.0 [26.0;29.0]	0.98	28.0 [26.0;29.0]	28.0 [26.0;29.0]	0.94	28.0 [26.0;29.0]	28.0 [26.0;29.0]	0.58
SCOPA-AUT	9.0 [6.0;14.5]	12.0 [7.0;19.3]	**0.03**	10.0 [6.0;17.0]	13.0 [7.50;18.0]	0.07	10.0 [6.0;15.0]	14.0 [10.0;22.0]	**<0.001**	11.0 [6.0;15.8]	17.0 [9.0;21.0]	**0.009**
Supine drop in SBP, mmHg	1.0 [−4.75;7.00]	2.0 [−3.0;10.0]	0.24	1.0 [−4.25;7.0]	3.0 [−2.0;12.0]	0.06	1.0 [−5.0;5.50]	8.0 [−0.250;13.3]	**0.011**	1.0 [−5.0;8.0]	4.50 [1.0;10.5]	0.04
Orthostatic SBP drop >10 mmHg	18 (19%)	16 (25%)	0.44	21 (17%)	13 (33%)	0.07	18 (15%)	16 (39%)	**0.002**	28 (21%)	6 (24%)	0.60
Nonmotor Symptom Scale	8.0 [4.0;10.0]	8.0 [5.50;13.0]	**0.03**	7.50 [4.0;10.0]	10.0 [7.0;13.0]	**<0.001**	7.0 [4.0;9.0]	11.0 [9.0;15.0]	**<0.001**	8.0 [4.0;10.0]	12.0 [7.75;14.3]	**0.001**
Olfaction (UPSIT 0–40)	21.0 [16.0;26.0]	23.0 [19.0;26.0]	0.09	22.0 [18.0;26.0]	19.0 [15.0;25.5]	**0.03**	23.0 [19.0;28.0]	17.0 [15.0;21.0]	**<0.001**	22.0 [17.0;27.0]	20.5 [17.0;24.0]	0.28
**Treatment (daily dose), mg/d**												
Total levodopa equivalent	250 [129;400]	250 [100;400]	0.57	250 [100;400]	299 [187;456]	0.19	224 [100;400]	300 [200;470]	0.07	250 [100;400]	324 [174;512]	0.31
Dopamine agonist levodopa equivalent	74.3 [0;150]	0 [0;150]	0.12	0 [0;150]	143 [27.8;190]	**0.002**	37.1 [0;150]	0 [0;150]	0.58	25.7 [0;150]	70.0 [0;150]	0.70
Levodopa	0 [0;300]	0 [0;299]	0.79	0 [0;300]	81.3 [0;263]	0.714	0 [0;250]	200 [0;300]	**0.032**	0 [0;299]	150 [0;300]	0.42

Data are given as mean ± SD or *N* (%). *P* = *p* values from Fisher’s exact test for categories or Wilcoxon test for continuous measures. Statistical significance was set at *p* < 0.05. Significant differences are shown in bold.

*HAD* Hospital Anxiety and Depression scale, *MDS-UPDRS* Movement Disorder Society-Sponsored Revision of Unified Parkinson’s Disease Rating Scale, *MoCA* Montreal Cognitive Assessment, *NMSS* Non-Motor Symptoms Scale, *SBP* systolic blood pressure, *SCOPA-AUT* Scales for Outcomes in Parkinson’s Disease—Autonomic dysfunction; UPSIT, University of Pennsylvania Smell Identification Test (40-item version).

**Table 5:** Clinical and polysomnography evaluations in participants with PD, classified by sleep disturbances

	Insomnia		*p*	Excessive daytime sleepiness		*p*	Define RBD		*p*	Restless leg syndrome		*p*	Sleep disordered breathing		*p*
	No (*N* = 95)	Yes (*N* = 65)		No (*N* = 121)	Yes (*N* = 40)		No (*N* = 119)	Yes (*N* = 41)		No (*N* = 135)	Yes (*N* = 25)		No (*N* = 143)	Yes (*N* = 19)	
**Sleep disturbances**															
Insomnia	NA	NA	NA	46 (38%)	18 (45%)	0.58	50 (42%)	15 (37%)	0.58	50 (37%)	15 (60%)	**0.045**	56 (40%)	9 (47%)	0.45
Initiating sleep	NA	NA	NA	15 (12%)	5 (12%)	1	14 (12%)	7 (17%)	0.43	13 (10%)	8 (32%)	**0.006**	18 (13%)	3 (16%)	0.47
Maintaining sleep	NA	NA	NA	40 (33%)	16 (40%)	0.57	42 (35%)	14 (34%)	1	45 (33%)	11 (44%)	0.36	49 (34%)	7 (37%)	0.80
Excessive daytime sleepiness	22 (23%)	18 (28%)	0.58	NA	NA	NA	29 (24%)	11 (27%)	0.84	34 (25%)	6 (24%)	1	35 (25%)	5 (26%)	0.78
Epworth sleepiness scale	7.0 [4.0;10.0]	7.50 [5.0;11.0]	0.53	6.0 [4.0;8.0]	13.0 [11.0;14.3]	**<0.001**	7.0 [5.0;10.0]	7.0 [4.0;11.0]	0.83	7.0 [4.25;10.8]	8.0 [5.0;10.0]	0.43	7.00 [5.00;10.0]	8.00 [4.50;10.3]	0.82
Defined RBD	26 (27%)	15 (23%)	0.58	30 (25%)	11 (28%)	0.84	NA	NA	NA	33 (24%)	8 (32%)	0.46	37 (26%)	4 (21%)	1
RBDQ- HK total score	7.50 [2.00;15.0]	10.0 [3.25;17.8]	0.3	7.50 [3.0;16.5]	9.0 [4.0;15.5]	0.38	5.0 [2.0;10.0]	24.0 [16.0;37.0]	**<0.001**	8.0 [2.25;15.0]	11.5 [4.0;18.0]	0.17	8.00 [3.00;15.3]	7.00 [3.25;18.0]	0.84
Factor 1 of RBDQ-HK (dreams)	3.0 [1.0;7.0]	5.0 [3.0;9.0]	**0.04**	4.0 [1.0;8.0]	5.0 [2.50;8.0]	0.19	3.0 [1.0;6.0]	10.0 [4.0;15.0]	**<0.001**	4.0 [1.0;8.0]	5.50 [3.0;9.75]	0.15	4.00 [2.00;8.00]	3.00 [2.00;8.00]	0.69
Factor 2 of RBDQ-HK (behaviors)	4.00 [0;8.50]	2.0 [0;12.0]	0.96	4.0 [0;10.0]	4.0 [0;10.0]	0.58	2.0 [0;4.0]	14.0 [11.0;24.0]	**<0.001**	4.0 [0;10.0]	4.0 [0;12.0]	0.51	4.00 [0;10.0]	4.00 [2.00;12.0]	0.37
Restless legs syndrome	10 (11%)	15 (23%)	**0.05**	19 (16%)	6 (15%)	1	17 (14%)	8 (20%)	0.46	NA	NA	NA	22 (15%)	3 (16%)	1
**Polysomnography measures**															
Total sleep time (min)	390 [336;425]	342 [305;392]	**0.002**	363 [318;417]	370 [320;416]	0.76	374 [320;418]	349 [317;408]	0.33	365 [323;419]	342 [311;403]	0.12	368 [331;419]	318 [296;354]	**0.014**
Sleep efficiency (%)	81.5 [75.4;88.8]	78.0 [69.6;85.1]	**0.02**	80.6 [73.5;88.5]	79.9 [72.2;85.2]	0.43	81.1 [74.4;89.0]	78.6 [70.9;86.6]	0.17	81.0 [73.6;88.2]	78.5 [73.3;86.2]	0.57	81.0 [75.2;88.5]	72.3 [67.5;80.4]	**0.001**
Wake after sleep onset (min)	80.5 [52.5;118]	95.0 [67.5;127]	0.08	82.3 [52.9;120]	95.0 [69.3;124]	0.28	83.5 [51.8;119]	95.0 [63.0;135]	0.16	89.5 [57.0;122]	95.0 [65.0;125]	0.75	83.5 [53.0;118]	114 [82.8;157]	**0.025**
**Latency to (min)**															
Sleep onset	13.0 [7.00;22.0]	14.5 [9.50;25.0]	0.2	14.0 [8.00;27.0]	10.5 [6.50;18.0]	0.05	13.0 [7.50;22.0]	15.0 [9.50;34.0]	0.08	13.5 [8.0;23.3]	11.0 [5.0;19.0]	0.16	13.5 [7.75;23.0]	14.0 [8.0;25.8]	0.92
REM sleep	139 [76.0;197]	136 [87.0;201]	0.94	130 [80.0;180]	160 [103;241]	0.08	139 [84.3;200]	137 [69.0;197]	0.71	137 [80.5;214]	142 [66.0;164]	0.16	133 [80.0;195]	156 [77.3;234]	0.28
**Sleep stages, % of total sleep time**															
N1 sleep	4.90 [2.15;7.80]	5.20 [1.90;9.10]	0.57	5.20 [2.50;8.00]	2.75 [1.15;8.55]	0.09	4.80 [2.00;7.85]	5.20 [1.50;9.30]	0.85	5.10 [1.90;8.35]	4.80 [3.50;9.30]	0.81	4.70 [1.40;7.75]	6.30 [4.95;12.3]	**0.008**
N2 sleep	53.9 [46.0;60.7]	49.6 [43.3;60.2]	0.28	51.4 [43.6;60.1]	57.7 [47.4;64.0]	**0.04**	52.4 [45.4;61.8]	52.9 [40.5;58.8]	0.24	52.9 [45.1;60.8]	49.6 [43.4;59.2]	0.38	51.5 [43.9;60.0]	58.2 [45.9;61.6]	0.20
N3 sleep	23.8 [17.6;30.3]	26.5 [18.7;32.9]	0.15	24.4 [17.6;31.2]	24.4 [19.2;31.4]	0.95	24.4 [18.2;30.9]	24.3 [18.3;31.5]	0.86	24.4 [18.2;30.9]	27.0 [18.7;33.6]	0.64	24.5 [18.8;31.6]	21.5 [12.4;28.2]	0.07
REM sleep	14.9 [11.5;19.8]	15.9 [8.90;20.4]	0.85	16.5 [11.6;21.4]	12.8 [8.30;16.9]	**0.009**	14.6 [9.65;19.5]	16.6 [12.2;22.5]	0.15	15.3 [11.1;20.4]	15.3 [10.7;18.2]	0.83	15.6 [11.5;20.1]	8.90 [7.95;19.1]	0.09
**Sleep fragmentation**															
Fragmentation index (arousals + awakenings/h	10.5 [6.71;14.7]	11.7 [7.90;16.9]	0.16	11.0 [7.16;15.2]	11.8 [7.59;19.2]	0.30	11.7 [7.61;16.9]	9.32 [5.79;14.6]	0.08	11.0 [7.0;15.5]	12.2 [7.90;18.7]	0.29	10.5 [6.81;14.5]	17.8 [14.1;25.0]	**<0.001**
Apnea-hypopnea/h	3.30 [0.80;8.70]	4.20 [1.50;8.40]	0.60	3.60 [0.90;8.40]	3.10 [0.95;7.80]	0.66	3.70 [0.85;8.05]	3.60 [0.90;9.60]	0.95	3.70 [0.85;8.05]	3.30 [1.10;9.20]	0.74	2.70 [0.70;6.80]	21.9 [17.9;30.1]	**<0.001**
Apnea-hypopnea index > 15/h	9 (10%)	9 (14%)	0.45	13 (11%)	5 (14%)	0.78	14 (12%)	4 (10%)	1	15 (11%)	3 (12%)	1	NA	NA	NA
Periodic leg movements/h	0.40 [0;3.08]	0.250 [0;4.85]	0.91	0.30 [0;3.60]	0.20 [0;3.70]	0.78	0 [0;3.10]	0.70 [0;8.60]	0.15	0 [0;2.60]	3.1 [0.70;12.3]	**0.003**	0.40 [0;3.70]	0 [0;1.85]	0.27
PLM > 15/h	14 (15%)	8 (12%)	0.82	18 (15%)	4 (10%)	0.60	13 (11%)	9 (22%)	0.11	17 (13%)	5 (20%)	0.35	20 (14%)	2 (11%)	1
REM without atonia (%)	8.20 [0.75;37.0]	10.0 [2.78;25.0]	0.72	10.0 [1.10;34.0]	8.00 [0;21.5]	0.42	6.0 [0;13.8]	40.8 [23.8;64.5]	**<0.001**	9.0 [0.50;30.5]	10.0 [3.0;33.0]	0.80	9.00 [1.08;33.3]	11.5 [0.93;26.1]	0.97

Data are given as Median [Q1–Q3] or *N* (%) (for categorical variables). *P* = *p* values from Fisher’s exact test for categories or Wilcoxon test for continuous measures. Statistical significance was set at *p* < 0.05 and shown in bold.

*NA* not appropriate, *PLM* periodic leg movements, *RBD* REM sleep behavior disorder, *RBDQ- HK* RBD questionnaire-Hong Kong, *vPSG* videopolysomnography.

### Factors associated with excessive daytime sleepiness

In the PD group, participants with EDS ([40/161],25% ; missing data = 1) did not differ in terms of age, sex and body mass index compared to participants without EDS. They had higher disability scores at the Part I, Part II, and total MDS-UPDRS, psychiatric (anxiety, depression and apathy) scales and NMSS, lower olfaction performances and took higher doses of dopamine agonists (Table 4). Total sleep time, sleep efficiency, latency and fragmentation did not differ between participants with and without EDS, but REM sleep percentage was lower in the group with EDS, to the profit of N2 percentage (Table 5). Notably, EDS was not associated with insomnia, RBD, RLS, SDB and abnormal PLM index.

### Factors associated with REM sleep behavior disorder

On average, PD participants with video-polysomnography-defined RBD ([41/160],25%; missing data = 2) were older, had higher body mass index and disability scores at the MDS-UPDRS (Part I, Part II, total, but not Part III and axial motor score) and HAD-depression scale, more markers of dysautonomia (drop in systolic blood pressure, orthostatic hypotension, SCOPA-AUT, NMSS), lower olfactory (but not cognitive) performances than participants without RBD and took higher doses of levodopa (Table 4). As expected, the RBD-HK total score and the percentage of REM sleep without atonia were higher in PD participants with than without definite RBD, and no further differences in sleep measures were found (Table 5). RBD was not associated with insomnia, EDS, RLS, SDB and abnormal periodic leg movements.

### Factors associated with restless leg syndrome

In the PD group, participants with RLS ([25/160], 16% ; missing data=2) had longer disease duration (but not different age, sex and body mass index) than participants without RLS (Table 4). They had more frequent markers of nonmotor symptoms, as indicated by higher scores at the MDS-UPDRS Part I, SCOPA-AUT and NMSS, but not different motor scores, doses of dopaminergic agents, psychiatric symptoms (anxiety, depression and apathy) scores, cognitive and olfactory performances and hypotension measures. Sixty percent of participants with RLS had insomnia (Table 5). Additionally, RLS was associated with difficulties initiating (but not maintaining) sleep, and higher periodic leg movement index, but not with EDS, RBD, SDB and other sleep measures.

### Factors associated with sleep-disordered breathing

Participants with SDB ([19/162], 12%; no missing data) were older (66.7 ± 7.0 years old) than participants without SDB (61.9 ± 9.3 years old; P = 0.013). No other differences in clinical parameters were observed (data not shown). SDB was not associated with insomnia, EDS, RBD, RLS, or abnormal periodic leg movements. Total sleep time and sleep efficiency were decreased, wakefulness after sleep onset, N1 percentage and fragmentation index were increased in participants with compared to without SDB.

## Discussion

Sleep disturbances were frequent (71%) and often combined in this systematic evaluation of sleep problems in a large, controlled group of participants with early stage PD with less than 4 years of disease duration. Insomnia (mainly difficulties maintaining sleep, not initiating it) was the most common, followed by RBD, EDS and marginally RLS, and they were more common than in controls, in contrast to SDB, which was as prevalent in PD participants as in controls. Almost half of the patients combined at least two types of sleep disturbances. Although each type of sleep disturbance could be combined with any other type in a patient, significant associations between sleep disorders were restricted to insomnia and RLS, whereas RBD, EDS and SDB were independent problems.

More than two-thirds of the participants with early stage PD had at least one type of sleep disturbance, and nearly half had multiple sleep disturbances. In the Parkinson’s Progression Markers Initiative (PPMI), 44.5% of 218 participants with early stage PD reported on questionnaires at least one type of sleep disturbance, including insomnia, EDS or probable RBD (as polysomnography was not performed) and 11.5% reported at least two types of sleep disturbances^6^. The higher figures in our series are probably explained by a greater number of sleep disturbances’ types (including RLS and SDB which were not studied in the PPMI), greater sensitivity and accuracy of face-to-face interviews with sleep specialists regarding the diagnosis of insomnia (vs. questionnaires in the PPMI^6^ and Norwegian ParkWest study in early stage PD^3,6^) and the high sensitivity of video-polysomnography for diagnosing definite RBD and SDB. The variety of types of sleep disturbances and their multiple combinations illustrates the complexity of sleep disturbances in PD. If all types of sleep disturbances combinations were observed, none were more prevalent, except for the association of insomnia with RLS. The number of sleep disorders increased with disease duration and dysautonomia. These results were probably driven by the presence of RBD and EDS, which in simple comparison are associated with more frequent non-motor symptoms. It is important to note that participants with multiple sleep disorders had no higher motor or cognitive impairment. One may expect that combined sleep disorders would be associated with greater degeneration of non-dopaminergic neurons in the brainstem and diencephalon (where sleep and wake systems are located). This co-occurrence seems important to consider because it is relatively common even in subjects with early disease. One may assume that the more sleep disturbances there are, the more they affect the quality of life and the more complex they are to manage, necessitating referral to sleep specialists. In addition, it would be interesting to assess long-term cognitive and motor outcomes in relation to the initial burden of sleep-related symptoms.

Insomnia was the most common sleep problem in PD and mostly consisted into difficulties maintaining sleep rather than initiating it (as in previous cohorts based on questionnaires in early^3,6^ and advanced PD^12^). The prevalence of insomnia in the general population varies from 4% to 48%^15^ depending on the definition used. Using the ICSD-3 criteria, including daytime impairment and a duration component, 21% of controls had insomnia, which is consistent with findings in other studies using similar criteria^16^. The prevalence of insomnia in PD (41%) was higher than the 21% to 31% previously reported^3,6^. The clinical factors associated with insomnia in PD are diverse and are likely to evolve over time as the disease progresses. In our study, insomnia was most common among women and participants with RLS (who also have higher rates of insomnia in the general population too)^17,18^, with more frequent non-motor symptoms and distressing dreams. Notably, insomnia was not associated with higher motor disability and doses of dopaminergic agents in this early stage of PD, in contrast to advanced PD, probably because our participants may not suffer from motor fluctuations with severe bradykinesia, tremor, and dystonia that disturb the night in advanced PD^1^. The lack of an association between insomnia and psychiatric features, such as depression or anxiety in our study was unexpected, especially given the known association in the general population^19^ and in few studies in PD^3,20^. However, a single study of de novo PD patients^3^ has shown higher mean depression scores (without exceeding the depression threshold) in PD participants with than without insomnia. In contrast, baseline insomnia was not associated with depression and anxiety at 6 months follow-up in another large population^21^. In our study, PD participants received dopaminergic treatment, which likely improved the anxiety-depression score^22^. In addition, participants with insomnia had lower total sleep time (median 342 min, i.e. 48 min less than those without insomnia) and sleep efficiency on sleep recordings, which supports their complaints with objective findings. The co-occurrence of insomnia with objectively short sleep duration (<6 h) represents the most biologically severe phenotype of insomnia disorder, as it confers higher health risks^23,24^. It further supports the concept of an organic origin in early PD.

RBD was the second more frequent sleep disorder in early stage PD, as it was found in one fourth of the sample. It was found exclusively in the PD population and never in the control group, confirming its specificity in synucleinopathies. It was associated with a distinct and more severe phenotype even in early stage PD. Definite RBD was associated with older age and higher body mass index (but not with male gender), more severe nonmotor features (mostly orthostatic hypotension, depression and hyposmia, but not cognitive impairment or apathy), higher subjective (but not objective) motor disability and higher doses of levodopa. Our results are consistent with many of those obtained in more advanced PD patients with definite RBD^25,26^ and in early stage PD patients with probable RBD^27^. The absence of cognitive impairment in early stage PD patients with RBD is of interest because the presence of RBD during the first 4 years of PD predicts earlier development of dementia in PD^28,29^. Nevertheless, this result should be considered with caution, since only PD patients with an MMSE greater than 26 were included in the cohort. This large series also indicates that at this early stage of PD, definite RBD is not associated with other sleep disorders or with specific polysomnographic abnormalities including periodic leg movements or REM sleep time (to the exception of REM sleep without atonia, which contributes to define RBD).

Excessive daytime sleepiness (defined as an ESS score greater than 10) was found in a quarter of PD participants and in 8% of controls. This prevalence in controls is consistent with the 8.5% to 22.2% prevalence of EDS in epidemiologic studies of the general population^30–32^. In PD, EDS may be caused by multiple factors, including greater spread of non-dopaminergic neurodegeneration within the brainstem and hypothalamus^5^, use of dopamine agonists and depression. Here, EDS was associated with increased severity and frequency of anxiety, depression and apathy symptoms, with lower olfaction and higher dopamine agonists dose. Notably, in contrast with sleepiness in the general population, EDS was not associated with SDB (and apnea-hypopnea index), with RLS (and periodic leg movements) or with insomnia (and total sleep time and sleep efficiency), as we earlier found in patients with advanced PD^33^. These results indicate that EDS is more often the consequence of central determinants (including psychiatric and neurologic symptoms as well as sedative effects of dopamine agonists) than sleep disturbances in PD^5,34–36^. Circadian causes of EDS were however not examined here.

Sleep-disordered breathing is common in middle-aged men in the general population and favored by narrow instable upper airways. Here only 12% of PD participants had SDB, a frequency similar to that of SDB in the control population^37^, probably reflecting the middle-old age of the samples rather than an intrinsic disorder related to early stage of PD. These results confirm, at an early stage of PD and in a large sample, the minimal importance of SDB in PD that we and others have observed in more advanced PD^38,39^. Indeed, most studies observed a similar^38^ or lower prevalence^39,40^ of sleep apnea in PD patients than in controls. In addition, SDB mainly present in a mild form, which is consistent with a previous study describing milder manifestations and lower oxyhemoglobin desaturations in PD with OSA compared to controls^41^. Within the PD participants, SDB was associated with older age and a more instable sleep, including increased N1 sleep and arousal index (which is expected as apnea and hypopnea cause arousals and lighter sleep), but also lower total sleep time and efficiency, which was less expected in SDB. However, SDB was not associated with any worsening of motor and nonmotor symptoms (including dysautonomic, cognitive and psychiatric symptoms, as well as EDS or insomnia), suggesting that it had no impact on the neurological disorder and had no clinical consequences.

This study has some limitations, including a monocentric design (which however warrantees the homogeneity of measures), in an expert center for PD (leading to possible recruitment bias towards more severe cases compared to a community study), a possible first night effect for video-polysomnography and the influence of treatments. However, this is representative of a standard real practice, which was applied to both PD and control participants. Strengths of the study include its systematic aspect (patients were recruited for early PD, not because they had sleep problems), which provides clear figures on the prevalence of all sleep disorders, especially those based on clinical plus polysomnography criteria. Large sample size, adjustment for confounding factors (age, sex, apnea hypopnea index) and a control group give a more robust result. The systematic, extensive collection of motor and nonmotor symptoms and signs, as well as the results of cognitive tests and video-polysomnography allow making new association studies. All in all, it provides a comprehensive analysis of all sleep problems at an early stage of PD.

In conclusion, sleep disturbances are an early problem in PD patients because they are frequent (two third of patients are affected) and complex (combination of sleep disorders is frequent). Their determinants are more organic than psychological, even at this early stage when bradykinesia, dystonia and tremor do not yet disturb sleep. They are likely underdiagnosed and their impact on daily life is underestimated. The timely and comprehensive clinical evaluation of sleep disorders in early PD patients is critical to improve management strategy and achieve a more personalized medicine. Sleep recording and sleep expert evaluation should also be considered in case of complex and severe sleep disorders. It remains to be determined whether sleep disorders (in addition to RBD) at this early stage influence motor and cognitive development of PD in the long term.

## Methods

### Participants

Participants were recruited from November 2014 to March 2021 as part of the ICEBERG longitudinal cohort (ClinicalTrials.gov: NCT02305147), which focuses on clinical and radiological trajectories in early PD. The inclusion criteria comprised an age between 18 and 75 years, presenting a PD according to the UK Parkinson’s Disease Society Brain Bank criteria, no or minimal cognitive disturbances (defined as a Mini-Mental State Examination [MMSE] score greater than 26/30), and a disease course lower than 4 years, defined as the period from the diagnostic by a neurologist to inclusion time. The usual treatment of participants was not stopped during evaluations. The inclusion criteria for healthy participants comprised the absence of present or past neurological or psychiatric disorder (as assessed after interview and clinical examination by a neurologist) and balanced for age and sex with participants with PD. All participants signed a written informed consent to take part in the study, which had been approved by the local ethics committee (CPP- Ile de France-Paris 6, RCB 2014-A00725-42). Healthy participants received a compensation for taking part in the study.

### Clinical assessment

We focused on the clinical examination and video-polysomnography at baseline. Interview, questionnaires and clinical examinations at baseline included: i) motor evaluation using the Movement Disorder Society Unified Parkinson’s Disease Rating Scale (MDS-UPDRS) Part 1 (nonmotor disability in daily life), Part II (motor disability in daily life), Part III (formal measure of motor disability measured by a neurologist in OFF-drug conditions) total score, and axial motor score (MDS-UPDRS III.1-9-10-12-13 sub score) ; ii) cognitive and neuropsychiatric evaluations including the Frontal Assessment Battery (FAB), Montreal Cognitive Assessment score (MoCA) (range :0–30, norms>26),, Mini Social and Emotional Assessment (miniSEA), Starkstein apathy scale (range :0–42, norms<15), and Hospital Anxiety and Depression Scale (HAD) (range :0–21, norms<7 for each part); iii) the evaluation of autonomic symptoms and signs using the SCOPA-AUT scale for autonomic dysfunction, Non Motor Symptom Scale (NMSS), systolic blood pressure drop measured from supine position (10 min) to supine position after 1 min, and orthostatic drop defined by a systolic arterial pressure drop greater than 10 mmHg measured one minute after standing up based on the study by Postuma^42^; and iv) olfaction performance using the University of Pennsylvania Smell Identification Test (UPSIT; 40-item version).

### Sleep evaluations

Sleep specialists (PD, SLS, IA) performed a systematic face-to-face interview about sleep disturbances. Participants completed the REM sleep behavior disorder (RBD) questionnaire from Hong Kong (RBDQ-HK) and the Epworth sleepiness scale (ESS). All subjects (patients and controls) were explored by night-time video-polysomnography in the Sleep Disorders Unit of the Pitié-Salpêtrière hospital, Paris, France. The recordings included three (F1/A2, C3/A2, O1/A2) to eight (F1/A2, C3/A2, O1/A2, T3/A2, F2/A2, C4/A2, T4/A2, O2/A2) electroencephalogram (EEG) bipolar channels, two electrooculograms (EOG), surface electromyogram (EMG) of the chin and left and right tibialis anterior muscles, electro-cardiogram (EKG), airflow via nasal pressure and naso-oral thermistor, respiratory efforts (via thoracic and abdominal plethysmography), transcutaneous oxyhemoglobin, body position, tracheal sound (snoring detector), as well as synchronized infrared video and ambient sounds. Sleep neurologists scored sleep stages, arousals, periodic leg movements (PLM) and respiratory events according to international criteria^43^. The sleep fragmentation index was the sum of wake episodes plus arousals per hour of sleep. PLM index was considered as abnormal if greater than 15/h^44^. Sleep-disordered breathing (SDB) was defined as an apnea-hypopnea index greater than 15/h^44^. Insomnia, RLS, video-polysomnography-defined RBD and disorders of arousal were diagnosed according to the ICSD-3 criteria^44^. Insomnia was separated into difficulties in initiating sleep, maintaining sleep or both. Excessive daytime sleepiness was defined as an ESS score greater than 10/24.

### Statistical Analysis

Comparisons between PD and control groups were performed using Fisher’s exact test (categories) and Welsch’s *t* tests (continuous measures). Generalized Linear models were adjusted for age and sex to compare between-groups clinical measures, and adjusted for age, sex, and apnea-hypopnea index to compare measures from sleep questionnaires and video-polysomnography. Generalized Linear models with Bernoulli family and logit link were used for binary measures and Generalized Linear models with Gaussian family and identity link for continuous measures. Due to a lack of statistical power to use Generalized Linear models for binary measures, Fisher’s exact test only were performed when at least one group sample contained less than 5 subjects. Multiple testing was handled using Benjamini-Hochberg correction. Normality of residuals and heteroskedasticity were visually checked. Cook’s distances and hat values were computed to investigate potential influencers and outliers. Six variables were right-skewed and were square root transformed. They included PLM index, apnea-hypopnea index, percentage of N1 sleep, latency to sleep onset, RBDQ-HK total score, and Factor 2 of RBDQ-HK. A sensitivity analysis was performed by restricting the analysis to participants not taking antidepressants. Within the PD group, the Kruskal-Wallis test for numerical variables and Fisher’s exact test for categorical variables were used to compare three groups defined by the number of sleep symptoms (i.e., 0, 1, and >1 symptoms). To determine which groups differed from each other, post hoc comparisons were made using the pairwise Mann–Whitney–Wilcoxon test for numerical variables and the pairwise Fisher’s exact test for categorical variables, both followed by the Benjamini–Hochberg correction to account for multiple testing. A forward stepwise procedure using the AIC criterion was used to select measures most associated with an increase in the number of sleep disturbances using ordinal logistic regression. The clinical and video-polysomnography measures were compared according to sleep disorders using Fisher’s exact test (categories) and Wilcoxon test (continuous measures) without any adjustment for multiple comparisons because of their exploratory nature^45^. The UpSetR v1.4.0 package based on full dataset concerning the presence of five sleep disturbances was used for building Fig. 1. All statistical analyses were performed using R Studio software, version 4.2.0 from the R Foundation for Statistical Computing (Vienna, Austria). Statistical significance was set at *P* lower than 0.05.

### Reporting summary

Further information on research design is available in the Nature Research Reporting Summary linked to this article.

### Supplementary information

supplementary figure 1

reporting summary

## Data Availability

Anonymized data not published within this article will be made available by request from any qualified investigator.
