# Supplementary material for: Sleep disorders in Parkinson’s disease, an early and multiple problem
Source: NPJ Parkinsons Dis. 2024 Feb 29;10:46. doi: 10.1038/s41531-024-00642-0 (PMC10904863; doi:10.1038/s41531-024-00642-0)
Supplement: Supplementary file 1 — supplementary figure 1 [file 41531_2024_642_MOESM1_ESM.pdf]

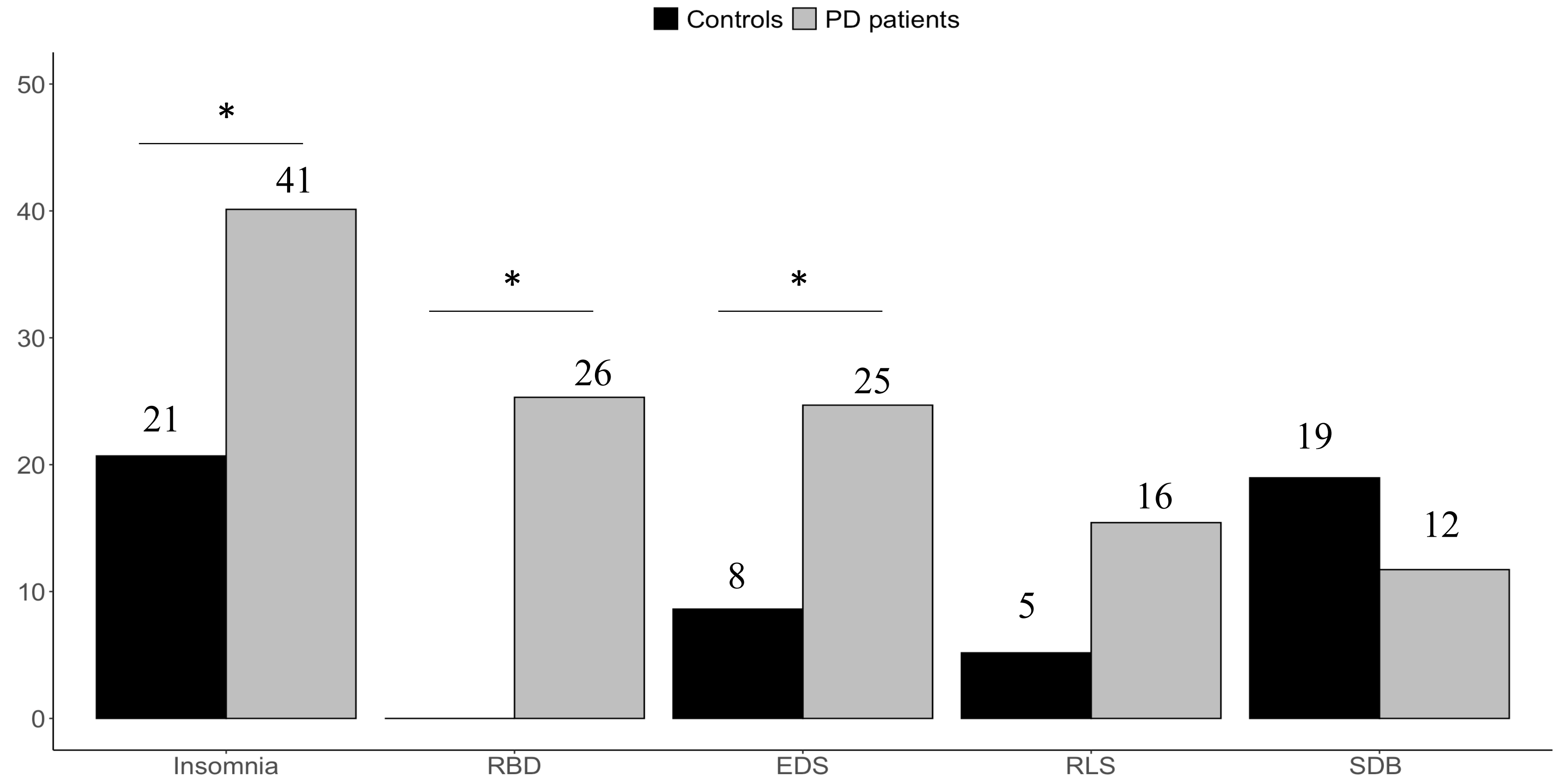

Supplementary Figure 1- Frequency of sleep disorders in participants with Parkinson's disease (grey columns) and controls (black columns) ; *P<0.05 for a between group difference

RBD, REM behavior disorder ; EDS, excessive daytime sleepiness; RLS, Restless legs syndrome; SDB, sleep-disordered breathing (apnea-hypopnea index > 15/h)
